# Csk controls leukocyte extravasation via local regulation of Src family kinases and cortactin signaling

**DOI:** 10.3389/fimmu.2024.1480152

**Published:** 2024-10-28

**Authors:** Rebekka I. Stegmeyer, Katrin Holstein, Kathleen Spring, Ilse Timmerman, Min Xia, Malte Stasch, Tanja Möller, Astrid F. Nottebaum, Dietmar Vestweber

**Affiliations:** ^1^ Department of Vascular Cell Biology, Max Planck Institute for Molecular Biomedicine, Münster, Germany; ^2^ BioOptic Service, Max Planck Institute for Molecular Biomedicine, Münster, Germany

**Keywords:** endothelial cells, VE-cadherin, ICAM-1, leukocyte extravasation, cortactin, Csk

## Abstract

C-terminal Src kinase (Csk) targets Src family kinases (SFKs) and thereby inactivates them. We have previously shown that Csk binds to phosphorylated tyrosine 685 of VE-cadherin, an adhesion molecule of major importance for the regulation of endothelial junctions. This tyrosine residue is an SFK target, and its mutation (VE-cadherin-Y685F) inhibits the induction of vascular permeability in various inflammation models. Nevertheless, surprisingly, it increases leukocyte extravasation. Here, we investigated whether endothelial Csk is involved in these effects. We found that the deficiency of Csk in endothelial cells augments SFK activation and the phosphorylation of VE-cadherin-Y685 but had no net effect on vascular leak formation. In contrast, the lack of endothelial Csk enhanced leukocyte adhesion and transmigration *in vitro* and *in vivo.* Furthermore, the silencing of Csk increased tyrosine phosphorylation of the SFK substrate cortactin. Importantly, the effects of Csk silencing on the increase in SFK activation, cortactin phosphorylation, and neutrophil diapedesis were all dependent on Y685 of VE-cadherin. Deletion of cortactin, in turn, erased the supporting effect of Csk silencing on leukocyte transmigration. We have previously shown that leukocyte transmigration is regulated by endothelial cortactin in an ICAM-1-dependent manner. In line with this, blocking of ICAM-1 erased the supporting effect of Csk silencing on leukocyte transmigration. Collectively, our results establish a negative feedback loop that depends on the phosphorylation of VE-cadherin-Y685, which recruits Csk, which in turn dampens the activation of SFK and cortactin and thereby the clustering of ICAM-1 and the extravasation of neutrophils.

## Introduction

Endothelial cells control vascular integrity and provide a barrier between the blood and the interstitium. The integrity of this barrier is compromised in many diseases by inflammatory mediators that trigger various molecular mechanisms. Some of these mechanisms target endothelial cell junctions, thereby stimulating plasma leakage, while others stimulate the ability of endothelial cells to interact with leukocytes and enable them to extravasate.

The recruitment of leukocytes is stimulated by cytokines that induce the expression of endothelial adhesion molecules such as selectins, VCAM-1, and ICAM-1, which, in concert with chemokines, mediate capture, rolling, crawling, and adhesion (1, 2). This then enables leukocytes to pass the endothelial barrier, which mainly occurs through endothelial cell junctions (3–5). The complex process of leukocyte diapedesis requires the coordinated action of adhesion molecules, the actin cytoskeleton, and cytosolic signaling molecules that affect junction integrity (6, 7). An important aspect of the diapedesis process is the formation of a docking structure that is formed by a ring of ICAM-1 molecules and filamentous actin surrounding interacting leukocytes (8–11). Cortactin, a protein that promotes actin assembly (12, 13), is involved in this recruitment of ICAM-1 and actin filaments (14) and supports neutrophil transmigration through endothelial cell monolayers (15, 16). These *in vitro* studies were confirmed *in vivo* based on intravital microscopy of neutrophil extravasation in cortactin gene-deficient mice (17).

The cytoplasmic tyrosine kinase Src is a central player in cell signaling and is implicated in the regulation of proliferation, survival, adhesion, and migration. Many of its first identified substrates are known to be important regulators of the actin cytoskeleton (18). Cortactin is one of them (19), and it has been shown that Src-mediated phosphorylation of cortactin supports its function in actin assembly (20). In agreement with this, three tyrosine residues of cortactin that are substrates for Src are required for the supportive role of cortactin in neutrophil diapedesis (15).

Endothelial junctions are also affected by Src and other members of the Src family kinases (SFKs). VEGF-A-induced vascular leakage was found to be inactivated in both Src^−/−^ and Yes^−/−^ mice, whereas deletion of Fyn had no such effect (21). Several cytosolic factors are known as SFK substrates, some of which affect cell adhesion at cell junctions and others regulate adhesion at sites of cell–matrix interactions (18). One important substrate at junctions is VE-cadherin, an adhesion molecule of dominant importance in the regulation of endothelial junctions. Different tyrosine residues in VE-cadherin have been reported to play a role in the regulation of junctions (22–24). Tyrosine 685 (Y685) has been identified as a direct and major target of Src (22, 25). A point mutation of this tyrosine in knock-in mice reduced the induction of vascular permeability *in vivo* (26–28). This tyrosine was not required for leukocyte extravasation, whereas mutating Y731 selectively affected neutrophil extravasation *in vivo*, but not vascular permeability induction (7, 26). Interestingly, a recent study revealed that gene inactivation of Src and Yes in endothelial cells *in vivo* had opposite effects on endothelial junction integrity, with Yes having a supportive role and Src functioning as a destabilizer (29).

C-terminal Src kinase (Csk) is a negative regulator of SFKs that phosphorylates an inactivating tyrosine (Y529) at the C-terminus of SFKs (30, 31). It targets all known SFKs and is an important negative regulator of this kinase family. Systemic gene inactivation leads to embryonic lethality in mice (32, 33). In addition to multiple developmental defects, one of the reasons for lethality is based on defects in vascular remodeling (34). In contrast to SFKs, which are lipid-anchored in plasma membranes via their N-terminal myristate and palmitate moieties, Csk lacks such fatty acyl modifications. Therefore, the recruitment of Csk to the membrane is thought to have an important regulatory function. Several scaffolding or membrane proteins have been identified as possible membrane anchors for Csk (30). In endothelial cells, one of them is VE-cadherin, and phosphorylated Y685 has been described as a docking site for the SH2 domain of Csk (35).

In this study, we have analyzed *in vitro* and *in vivo* whether Csk is relevant for the control of the barrier function of endothelial cells. We found that endothelial-specific gene inactivation of Csk caused elevated activation levels of SFK at junctions but did not affect vascular permeability. In contrast, Csk inactivation increased neutrophil adhesion and extravasation *in vivo*. This effect was accompanied by tyrosine phosphorylation of cortactin and was blocked upon gene inactivation of cortactin in endothelial cells. Thus, interference with the function of Csk augments leukocyte extravasation by increasing the activation of cortactin but has no influence on vascular permeability.

## Materials and methods

### Mice

All mice were bred under pathogen-free conditions in the animal facility of the Max Planck Institute for Molecular Biomedicine and were of the C57BL/6 genetic background. All experiments were carried out as approved by the local state authorities [Landesamt für Natur, Umwelt und Verbraucherschutz Nordrhein-Westfalen (LANUV)].

In order to generate an endothelial-specific conditional Csk knock-out mouse line, Csk^lox/lox^ mice (36) were mated with transgenic mice expressing a tamoxifen-inducible form of the Cre recombinase in vascular endothelial cells using a phage artificial chromosome (PAC) containing the *Pdgfb* gene (Pdgfb-iCreER mice) (37). The following primers were used for genotyping: CSK forward (5′-TGG GGT TGA ATG GTA TGA ACA CTC-3′) and CSK reverse (5′-TGC CAT GTG GAG AAG AGA ATC AGC-3′). This generated a 500-base-pair PCR product for wild-type (WT) mice and a 600-base-pair product for mice carrying the loxP sites (Csk^lox^).

These mice were further mated with cortactin knock-out mice (17) or with VE-cadherin-Y685F mice (26), both previously described by our group.

### *In vivo* skin permeability assay

Blood vessel permeability in the skin was analyzed by a modified Miles assay as previously described (38). For each assay, four to five 8- to 12-week-old Csk^lox/lox^ or Csk^iECKO^ mice were i.p. injected for 5 consecutive days with 2 mg tamoxifen (#T5648-5G, Sigma-Aldrich, St. Louis, MO, USA) in sunflower oil. Mice were weighed, and their dorsal skin was shaved. Evans blue dye (Sigma-Aldrich) was injected into the tail vein [10 µL/1 g body weight of a 1% solution in phosphate-buffered saline (PBS)]. A total of 15 min later, PBS, 90 ng mVEGF, or 175 ng histamine was injected intradermally into the dorsal skin. Another 30 mins later, the mice were sacrificed, skin areas were excised, and leaked dye was extracted with formamide for 5 days. The optical densities of the samples were measured at 620 nm using a spectrophotometer (UV-1900i; Shimadzu, Kyoto, Japan).

### Intravital microscopy of the cremaster muscle

Mice at 11–20 weeks of age were used. General anesthesia was administered using ketamine (125 mg/kg body weight) and xylazine (12.5 mg/kg body weight), and mice were closely monitored during anesthesia. Surgical preparation of the cremaster muscle and intravital microscopy were performed as previously described (26, 38, 39). Mice received an intrascrotal injection of 50 ng of recombinant IL-1β (Biomol, Hamburg, Germany) in 150 µL PBS. After 4 h, the cremaster muscle was prepared, and intravital images were recorded using an upright Axio Examiner microscope (Axiocam MRM, 40x Plan Neofluar, NA 1.30, Carl Zeiss, Oberkochen, Germany). For each animal, three to eight single unbranched postcapillary venules with a diameter of 20 to 30 µm were analyzed. Blood flow centerline velocity was measured using a dual-photodiode sensor system (Circusoft, Hockessin, DE, USA).

Recorded images and videos were analyzed using ImageJ. The leukocyte rolling flux fraction was determined as a percentage of total leukocyte flux. Leukocyte rolling velocity was determined in each vessel segment, and the total number of adherent leukocytes was analyzed for each vessel segment (100 µm) and is depicted per 10^4^ µm^2^ vessel surface area. Transmigrated cells were counted in an area extending 75 µm outside of each vessel over a distance of 100 µm vessel length (corresponding to 1.5 × 10^4^ µm^2^ tissue area). Newtonian wall shear rate (s^−1^) and mean blood velocity were determined as previously described (38).

### Cell culture

Human umbilical vein endothelial cells (HUVECs) were isolated as described (40) and cultured in an EBM-2 medium with SigleQuot supplements (Lonza, Basel, Switzerland) and used for experiments between passages 3 and 6 (Ethics Committee of Münster University Clinic, approval 2009-537-f-S). Primary endothelial cells from the lungs of Csk^iECKO^ mice were isolated and cultured as described (17). For Csk knock-out induction, murine primary endothelial cells were treated for 5 consecutive days with 1 µM (*Z*)-4-hydroxytamoxifen (#sc-3542A, Santa Cruz Biotechnology, Dallas, TX, USA) or equivalent amounts of solvent (ethanol) in the medium. Murine polymorphonuclear neutrophils (PMNs) were isolated from bone marrow by density gradient centrifugation using Histopaque 1077 and 1119 (Sigma-Aldrich). The granulocyte-containing phase was collected and washed twice in Hanks balanced salt solution, 25 mM HEPES (pH 7.3), and 10% fetal calf serum. PMNs were cultured overnight in Dulbecco’s modified Eagle medium, 20% fetal calf serum, 1% glutamine, 1% penicillin/streptomycin, and 10% WEHI-3B culture supernatant. Before experiments, human umbilical vein endothelial cells (HUVECs) and murine primary endothelial cells were treated with TNF-α (recombinant human TNF-α #300-01A, PeproTech, Cranbury, NJ, USA; recombinant murine TNF-α #315-01A, PeproTech).

### RNA interference

For the knock-down of Csk and VE-cadherin in HUVECs siRNAs, the following sequences were used: 5′-TACGCGCCTCATTAAACCAAA-3′ (target sequence for Csk siRNA, Hs_CSK_3, Qiagen, Valencia, CA, USA) and 5′-GGUUUUUGCAUAAUAAGCtt-3′ (sense strand for CDH5 siRNA, 10696, Ambion, Austin, TX, USA). Routinely, dishes with 70% confluent HUVECs were transfected with 40 nM (Csk) or 20 nM (VE-cadherin) of siRNA using Lipofectamine RNAiMAX (#13778075, Invitrogen, Carlsbad, CA, USA), according to the manufacturer’s instructions.

For *in vitro* RNA interference with cortactin in murine endothelial primary cells, the following sequences were used: 5′-GAGAUGUGCUAGUGGCUUATT-3′ (sense strand for cortactin siRNA, Mm_Cttn_5, Qiagen) and 5′-CCAACAUAGAAAUGAUUGATT-3′ (sense strand for cortactin siRNA, Mm_Cttn_7, Qiagen). Primary endothelial cells at 100% confluence were transfected with 48 nM siRNA per reaction by nucleofection according to the manufacturer’s instructions (Amaxa Biosystems, Cologne, Germany).

As a negative control, an siRNA not targeting any known mammalian genes was used (AllStars negative control, 1027281, Qiagen). Experiments were carried out 72 h after transfection.

### Adenoviral infection of HUVECs

To investigate the role of tyrosine 685 of VE-cadherin, HUVECs were transfected with either a VE-cadherin-WT-EGFP or a VE-cadherin-Y685F-EGFP construct as described previously (26). Cells were incubated for 24 h and subsequently used in the experiments.

### *In vitro* transmigration assay

To analyze the transendothelial migration of murine PMNs across primary endothelial cell monolayers, 2 × 10^4^ cells were seeded on fibronectin-coated Transwell filters (6.5 mm, 5 µm pore size, Corning, New York, NY, USA), grown to confluence, and stimulated with 5 nM murine TNF-α for 4 h before the experiment; 2 × 10^4^ murine PMNs were allowed to transmigrate towards a 40 ng/mL CXCL-1 chemokine gradient (recombinant mouse CXCL-1/KC aa 29-96, #1395-KC-025, R&D Systems, Minneapolis, MN, USA) for 40 min. Transmigrated PMN numbers were determined using a CASY Cell Counter TT+ (Roche, Basel, Switzerland).

### *In vitro* adhesion of PMNs to endothelial cells

Murine primary endothelial cells were grown to confluence in 96-well plates coated with 100 μg/ml fibronectin and were subsequently stimulated with 5 nM TNF-α for 4 h. PMNs that were isolated 1 day prior were stained with Vybrant DiD dye (V22887, Invitrogen) according to the manufacturer’s instructions; 1 × 10^5^ PMNs in 200 µL medium [Hanks’ Balanced Salt Solution (HBSS), 25 mM HEPES (pH 7.0)] were added to each well and were allowed to adhere for 30 min at 37°C in 5% CO_2_. Adherent cells that did not firmly interact with the endothelial cell monolayer were removed by washing three to five times with pre-warmed PBS. The remaining PMNs were subsequently fixed with 4% paraformaldehyde (PFA), and images of each well were taken using an Axiovert 200M microscope (20x Plan Apochromat, NA 0.80, Carl Zeiss, Oberkochen, Germany). The number of adherent cells was determined using an ImageJ plug-in.

### Antibodies

Polyclonal rabbit antibodies VE42 against VE-cadherin (41) and C5 against VE-cadherin (42) and monoclonal antibodies mp685 against phosphorylated tyrosine 685 of murine VE-cadherin and mp731 against phosphorylated tyrosine 731 of VE-cadherin (26) and YN1/1.7 against ICAM-1 (43) have been described previously.

The following monoclonal antibodies were purchased: antibodies against phosphotyrosine (4G10, #05-321, Millipore, Billerica, MA, USA), antibodies against Csk (52, #610080, BD Biosciences, San Jose, CA, USA), antibodies against Src (GD11, #05-184, Millipore; or 327, #ab16885, Abcam, Cambridge, UK), antibodies against cortactin (4F11, #05-180, Millipore), antibodies against VE-cadherin (F-8, #sc-9989, Santa Cruz Biotechnology; or 75/Cadherin-5, #610251, BD Biosciences), antibodies against α-actin (B-12, #sc-166524, Santa Cruz Biotechnology), and antibodies against α-tubulin (B-5-1-2, #T6074, Sigma).

Furthermore, the following polyclonal antibodies were purchased: antibodies against Csk (H-75, #sc-1307, Santa Cruz Biotechnology), antibodies against the phospho-Src family (Tyr416) (#2101, Cell Signaling, Danvers, MA, USA), antibodies against phospho-SRC (Tyr529) (#44662G, Thermo Fisher, Waltham, MA, USA), antibodies against Src (SRC2, #sc-18, Santa Cruz Biotechnology), antibodies against phospho-Cortactin (Tyr421) (#C0739, Sigma), antibodies against phospho-Cortactin (Tyr421) (#4569, Cell Signaling), antibodies against VE-cadherin (C19, #sc-6458, Santa Cruz Biotechnology), and antibodies against GFP (#ab6673, Abcam).

Selective labeling of F-actin in immunostainings was achieved with phalloidin directly coupled to Alexa Fluor 647 (Invitrogen).

Horseradish peroxidase-coupled secondary antibodies were obtained from Dianova (Geneva, Switzerland), and IRDye680- and IRDye800-coupled secondary antibodies were purchased from LI-COR (Lincoln, NE, USA). Goat anti-mouse Alexa Fluor-coupled antibodies were acquired from Invitrogen.

### Immunofluorescence staining

Cells were cultured until they reached confluency on 8-well µ-slides (ibidi, Gräfelfing, Germany) as described in the “Cell culture” section. They were pretreated for 30 min with 1 mM Na_3_VO_4_, washed twice with PBS-MC, and fixed with 4% PFA for 10 min followed by permeabilization with 0.5% Triton X-100 for 5 min. Blocking was carried out for 1 h at room temperature (RT) with 3% bovine serum albumin (BSA), and antibody staining processes were performed for 1 h at RT in blocking buffer. Stained cells were either kept in PBS with 0.04% NaN_3_ or covered in a fluorescence mounting medium (Dako Omnis, Agilent Technologies, Santa Clara, CA, USA).

Z-stack images were acquired using a confocal laser scanning microscope (LSM 880, 63x Plan-Apochromat, NA 1.40, Carl Zeiss). ImageJ was used for the quantification of the phospho-Src signal. Briefly, the cadherin signal was used to create a mask of the endothelial cell junctions. The phospho-Src signal at the junctions was then calculated as a percentage of the total phospho-Src signal in the image.

ICAM-1 recruitment to transmigrating neutrophils was determined largely as described (17). Murine leukocytes were added to TNF-α-activated confluent primary endothelial cell monolayers. To this end, 2 × 10^5^ PMNs were added to each well of an 8-well µ-slide (ibidi, Gräfelfing, Germany) and allowed to transmigrate for 15 min. Wells were subsequently washed two times to remove unbound/non-transmigrating leukocytes and fixed with 4% PFA for 15 min. Further staining was performed corresponding to the abovementioned protocol for immunofluorescence staining processes of cells. Z-stack images were acquired using a confocal laser scanning microscope (LSM 780, 20x Plan-Apochromat, NA 0.8, Carl Zeiss). The mean fluorescence intensity of the ring-like ICAM-1-rich structures was determined in one single z-slice depicting the largest expansion of the ring using an ImageJ Macro. Values are depicted relative to the ones observed in control endothelial cells expressing cortactin (cells treated with solvent instead of tamoxifen).

### Immunoblot analysis and immunoprecipitation

Cells were lysed in lysis buffer (10 mM NaPi, 150 mM NaCl, 1% NP-40, 2 mM EDTA, 1 mM Na_3_VO_4_, 1x cOmplete EDTA-free Proteinase Inhibitor Cocktail, Roche, Basel, Switzerland). In order to detect phosphorylation signals after immunoprecipitations, cells were lysed in 20 mM Tris–HCl (pH 7.4), 150 mM NaCl, 2 mM CaCl_2_, 1.5 mM MgCl_2_, 1% Triton X-100, 0.04% NaN_3_, 1 mM Na_3_VO_4_, and 1x cOmplete. Lysates were centrifuged at 20,000 *g* at 4°C for 30 min before aliquots for immunoblot analysis were taken.

Murine lungs were homogenized using an ULTRA Turrax (IKA-Werke, Staufen, Germany) in radioimmunoprecipitation assay buffer containing 10 mM NaPi, 150 mM NaCl, 1% NP-40, 2 mM EDTA, 0.1% sodium dodecyl sulfate (SDS), 1% sodium deoxycholate, 1 mM Na_3_VO_4_, and 2x cOmplete. Tissue was lysed for 1 h at 4°C and centrifuged at 20,000 *g* at 4°C for 30 min before aliquots for immunoblot analysis were taken.

For immunoprecipitation of VE-cadherin, lysates were pre-cleared for 2 h at 4°C with protein A-Sepharose beads coated with an IgG control antibody. Subsequently, the immunoprecipitation was carried out for 2 h at 4°C with protein A-Sepharose beads coated with 4 µg (cell lysates) or 5 µg (lung lysates) of VE-cadherin C5 antibody. Immunocomplexes were washed five times with lysis buffer and analyzed by sodium dodecyl sulfate–polyacrylamide gel electrophoresis (SDS–PAGE).

Total cell or organ lysates were separated using SDS–PAGE in 8% gels and immunoprecipitated material in 10%–12% gels. Proteins were then transferred to a nitrocellulose membrane (Schleicher & Schuell, Keene, NH, USA) by wet blotting. For the detection of phosphorylated tyrosine residues, a blocking buffer containing 2% BSA and 200 µM Na_3_VO_4_ was used. Immunoblot signals were quantified using ImageJ or ImageStudio (LI-COR).

### Statistical analysis

Statistical significance was analyzed using a Mann-Whitney test, unpaired t-test, one-way analysis of variance (ANOVA) or two-way ANOVA for independent samples. The ROUT method was used to identify outliers, with a maximum false discovery rate of 0.1%. The GraphPad Prism 10 software was used for this analysis. Data are shown as mean ± standard error of the mean (SEM). P-values below 0.05 (*), 0.01 (**), 0.001 (***), and 0.0001 (****) were considered statistically significant.

## Results

### Gene inactivation of Csk leads to activation of SFKs and phosphorylation of VE-cadherin-Y685 but does not influence vascular permeability

**Figure 1:**
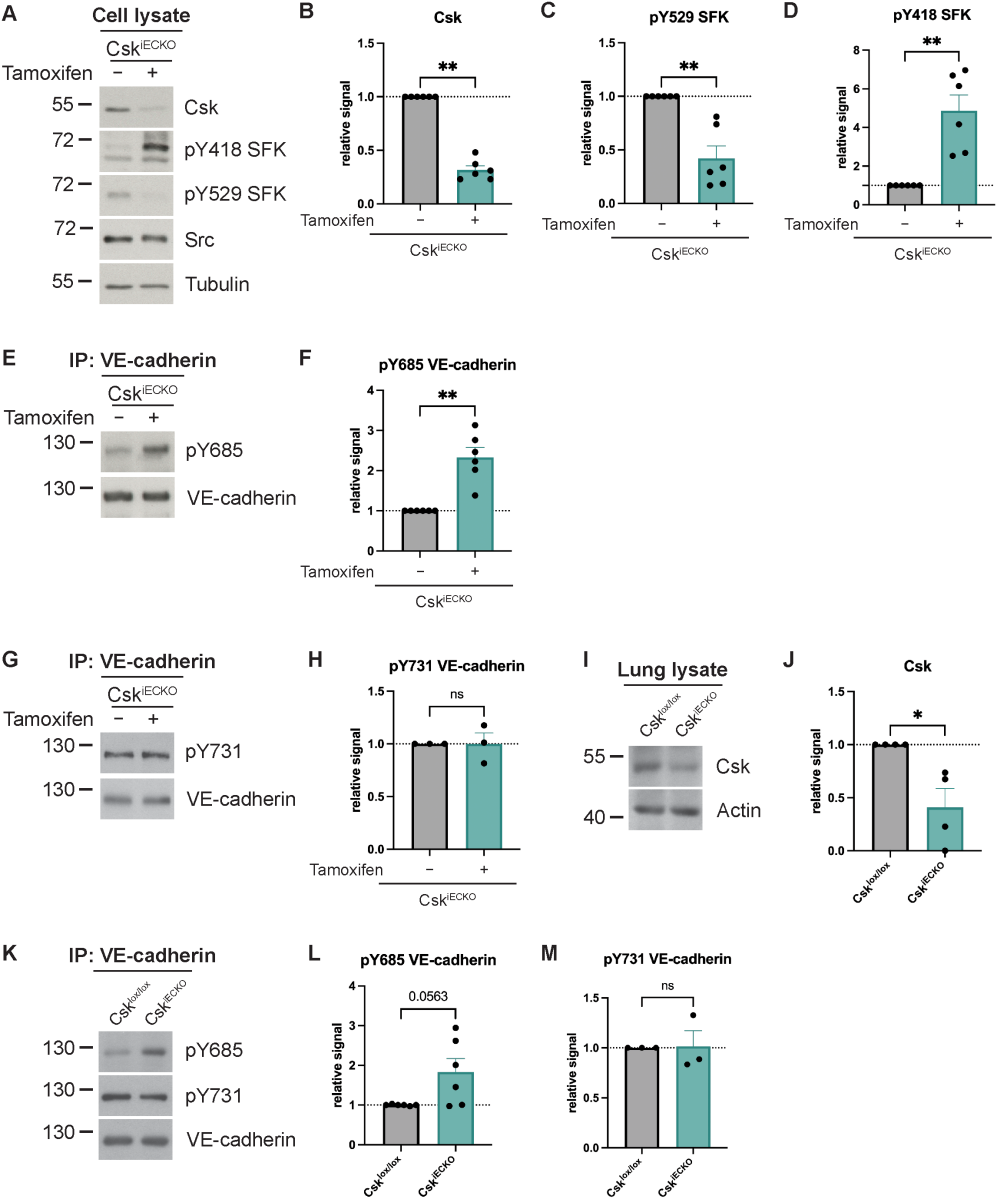
Csk deficiency increases Src activity and VE-cadherin phosphorylation *in vitro* and *in vivo*. **(A–D)** Total cell lysates from either solvent- (−) or tamoxifen-treated (+) endothelial cells isolated from the lungs of Csk^iECKO^ mice were immunoblotted and incubated with antibodies against Csk, phospho-SFK (Y418 and Y529), Src, and α- tubulin as a loading control **(A)** representative of six independent experiments. Molecular weight markers are indicated in kDa. The graphs depict the normalized relative Csk **(B)**, SFK-Y529 phosphorylation **(C)**, and SFK-Y418 phosphorylation **(D)** signal detected in the Western blots. The signal was normalized to total Src (for panels **C, D**), and α-tubulin and is presented relative to that of control cells, set as 1 (n = 6, mean ± SEM, **p < 0.01, Mann–Whitney test). **(E, F)** VE-cadherin was immunoprecipitated from either solvent- (−) or tamoxifen-treated (+) endothelial cells isolated from the lungs of Csk^iECKO^ mice. Blots were incubated with monoclonal antibodies for phosphorylated Tyr685 or phosphorylated Tyr731 of VE-cadherin **(E, G)** [representative of six **(E)** or three **(G)** independent experiments]. Molecular weight markers are indicated in kDa. The graphs depict the normalized relative VE-cadherin-Y685 phosphorylation **(F)** or normalized relative VE-cadherin-Y731 phosphorylation **(H)** signal detected in the Western blots. The signal was normalized to total VE-cadherin and is presented relative to that of control cells, set as 1 [n = 6 **(F)**, n = 3 **(H)**, mean ± SEM, ns = not significant, **p < 0.01, Mann–Whitney test]. **(I, J)** Csk^lox/lox^ and Csk^iECKO^ mice were i.p. injected with tamoxifen daily for 5 days. Total lung lysates were immunoblotted for Csk and actin (**I**, representative of four independent experiments). Molecular weight markers are indicated in kDa. The graphs depict the normalized relative Csk **(J)** signal detected in the Western blots. The signal was normalized to actin and is presented relative to that of Csk^lox/lox^ mice, set as 1 (n = 4, mean ± SEM, *p < 0.05, Mann–Whitney test). **(K–M)** Csk^lox/lox^ and Csk^iECKO^ mice were i.p. injected with tamoxifen daily for 5 days. VE-cadherin immunoprecipitates of lung lysates were immunoblotted for phosphorylated Tyr685 and Tyr731 of VE-cadherin and VE-cadherin [**K**, representative of six (pY685 VE-cadherin) or three (pY731 VE-cadherin) independent experiments]. Molecular weight markers are indicated in kDa. The graphs depict the normalized relative VE-cadherin-Y685 phosphorylation **(L)** or VE-cadherin-Y731 phosphorylation **(M)** signal detected in the Western blots. The signal was normalized to total VE-cadherin and is presented relative to that of Csk^lox/lox^ mice, set as 1 [n = 6 **(L)**, n = 3 **(M)**, mean ± SEM, ns = not significant, Mann–Whitney test].

In order to investigate the role of Csk in the control of endothelial barrier function, we generated Csk inducible endothelial-specific KO mice (Csk^iECKO^) by breeding Csk^lox/lox^ mice (36) with PDGFb-CreERT2 mice (37) (**Supplementary Figure 1A**). First, we analyzed the effect of Csk gene inactivation on the activation of SFKs. To this end, primary lung endothelial cells were isolated from Csk^iECKO^ mice and treated in culture with and without tamoxifen. As shown in **Figures 1A, B**, Csk expression was strongly reduced by 70% (± 6%) upon tamoxifen treatment. This was accompanied by a strong decrease in the phosphorylation of the inactivating tyrosine 529 residue of SFKs and a strong increase in the activating pY418 signal (**Figures 1C, D**). In agreement with this, phosphorylation of Y685 of VE-cadherin was also clearly increased, which is known to be an SFK target (**Figures 1E, F**). In contrast, no effect was seen for the phosphorylation level of Y731 (**Figures 1G, H**), which we have shown before to be constitutively phosphorylated. Dephosphorylation of this residue is involved in the leukocyte diapedesis step (26).

This result was verified by analyzing lung lysates prepared from Csk^lox/lox^ and Csk^iECKO^ mice after treating the mice daily for 5 days with tamoxifen. As shown by immunoblotting (**Figure 1I**), Csk expression was strongly reduced by 70% (± 19%) (**Figure 1J**). In agreement with our *in vitro* results, the phosphorylation level of Y685 of VE-cadherin was clearly increased (**Figures 1K, L**), while the phosphorylation level of Y731 of VE-cadherin was unaffected (**Figures 1K, M**).

**Figure 2:**
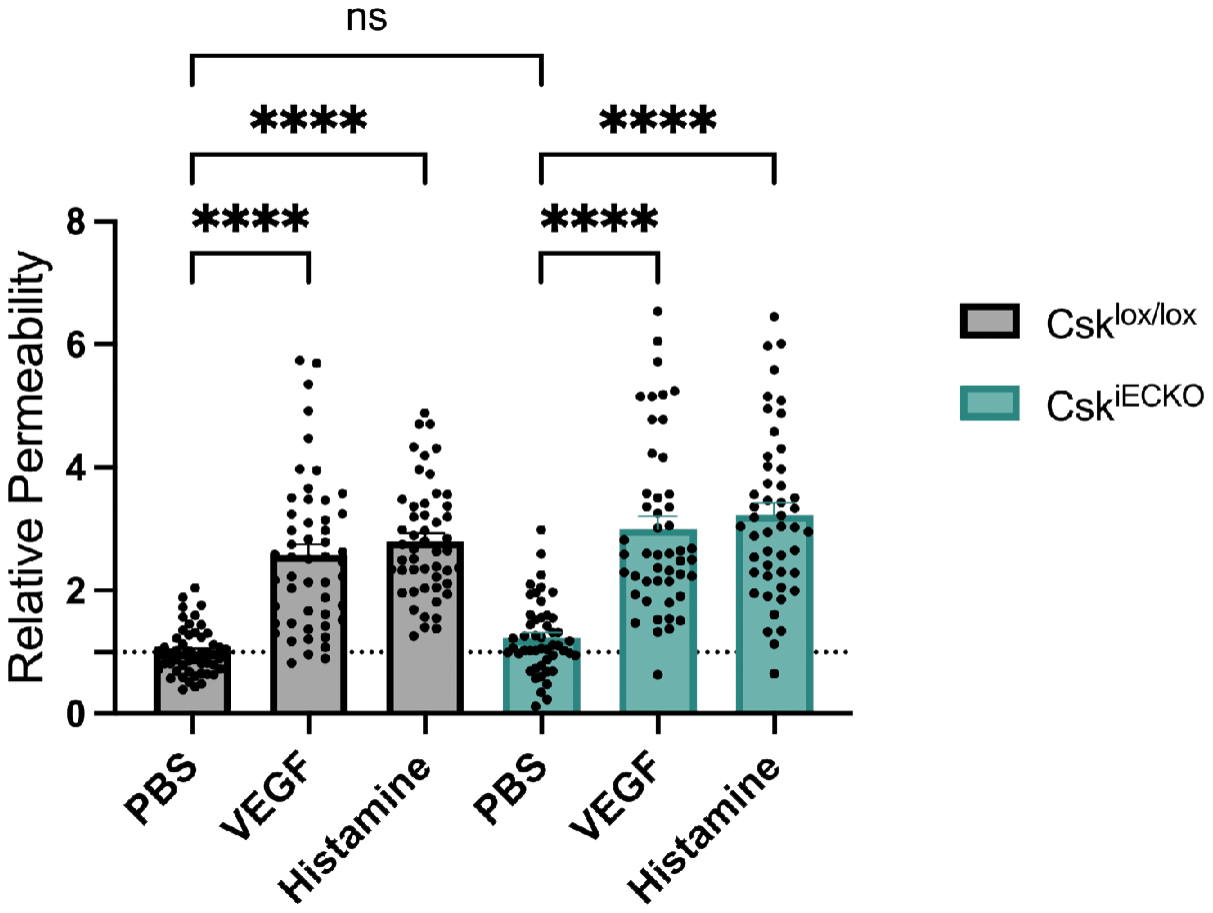
Csk deficiency does not increase vascular permeability. Csk^lox/lox^ and Csk^iECKO^ mice were i.p. injected with tamoxifen daily for 5 days. Afterward, mice were i.v. injected with Evan’s blue, followed by intradermal injection of phosphate-buffered saline (PBS), VEGF, or histamine (as indicated) 15 min later. After 30 min, the mice were sacrificed, and the dye was extracted from the skin biopsies and quantified. The graph depicts the relative permeability. The values are presented relative to that of the PBS-treated Csk^lox/lox^ mice, set as 1 (Csk^lox/lox^ n = 51 and Csk^iECKO^ n = 49, mean ± SEM, ****p < 0.0001, two-way ANOVA).

Next, we analyzed whether Csk gene inactivation would influence vascular permeability (**Figure 2**). To this end, we performed Miles assays with Csk^lox/lox^ and Csk^iECKO^ mice. We found that local intradermal injections of histamine or VEGF in mice that had been i.v. injected with Evans blue induced vascular leaks in Csk^lox/lox^ to the same extent as in Csk^iECKO^ mice (**Figure 2**). Furthermore, the baseline levels of vascular permeability as we determined them upon intradermal injection of PBS were also similar in mice of both genotypes (**Figure 2**). Thus, endothelial-specific gene inactivation of Csk neither increases baseline levels of vascular permeability nor augments the effects of histamine or VEGF on vascular leak formation. At first glance, this may appear surprising since an increase in VE-cadherin-Y685 phosphorylation is known to correspond to an increase in vascular leakage. However, since it has been recently shown that activation of Src and Yes has opposite effects on endothelial barrier integrity with respect to plasma leaks (29), inactivation of Csk may cause opposite and compensating effects on junction integrity via these two SFK family members.

### Gene inactivation of Csk leads to increased neutrophil extravasation

**Figure 3:**
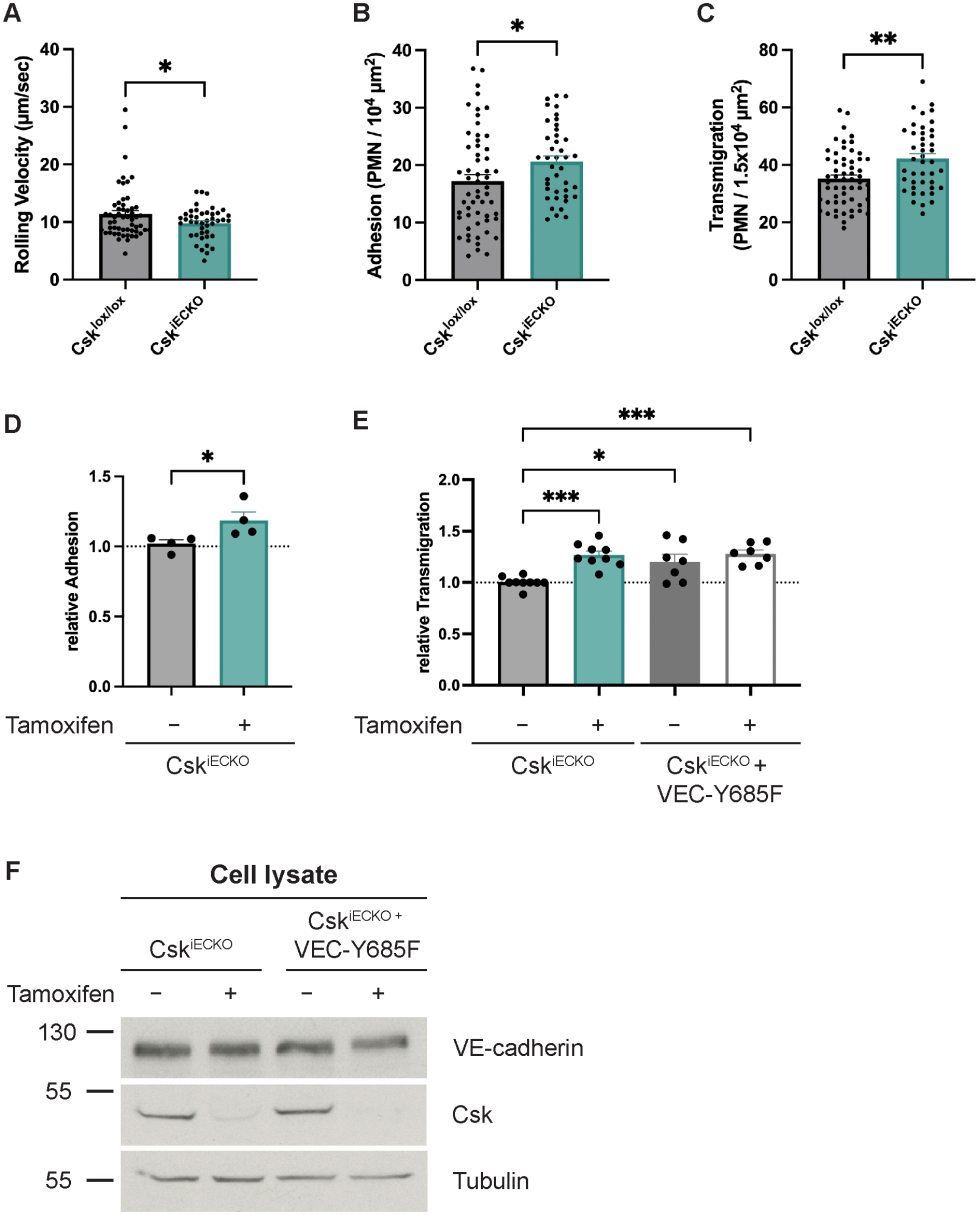
Deficiency of Csk increases leukocyte transmigration *in vivo* and *in vitro*. **(A–C)** Csk^lox/lox^ and Csk^iECKO^ mice were i.p. injected with tamoxifen daily for 5 days. The graphs depict rolling velocity **(A)**, adherent **(B)**, and extravasated **(C)** leukocytes in cremaster tissue of mice stimulated intrascrotally with IL-1β for 4 h before intravital microscopy (Csk^lox/lox^: n = 6 mice, n = 57 vessels; Csk^iECKO^: n = 5 mice, n = 43 vessels; mean ± SEM, *p < 0.05, **p < 0.01, t-test). **(D)** Adhesion of murine neutrophils to a TNF-α-stimulated monolayer of either solvent- (−) or tamoxifen-treated (+) endothelial cells isolated from the lungs of Csk^iECKO^ mice. The adhesion rate is presented relative to that of control cells, which was set as 1. One data point represents the mean adhesion determined in one experiment (n = 4, *p < 0.05, mean ± SEM, t-test). **(E)** Transmigration of murine neutrophils toward the chemokine CXCL-1 through a TNF-α-stimulated monolayer of either solvent- (−) or tamoxifen-treated (+) endothelial cells isolated from the lungs of Csk^iECKO^ or VE-cadherin-Y685F/Csk^iECKO^ mice. The transmigration rate is presented relative to that of control cells, which was set as 1. One data point represents the mean transmigration determined in one experiment [n = 9 (Csk^lox/lox^ and Csk^iECKO^) or n = 7 (VCY685F/Csk^lox/lox^ and VCY685F/Csk^iECKO^), mean ± SEM, *p < 0.05, ***p < 0.001, one-way ANOVA]. **(F)** Total cell lysates from either solvent- (−) or tamoxifen-treated (+) endothelial cells isolated from the lungs of Csk^iECKO^ or VE-cadherin-Y685F/Csk^iECKO^ mice were immunoblotted for Csk, VE-cadherin, and α-tubulin (representative of nine experiments). Molecular weight markers are indicated in kDa.

The lack of an effect of Csk deficiency on vascular permeability prompted us to test whether Csk is relevant for leukocyte extravasation *in vivo*. To this end, we stimulated tamoxifen-treated Csk^lox/lox^ and Csk^iECKO^ mice i.s. with IL-1β for 4 h before preparing the cremaster muscle for intravital microscopy and analyzing neutrophil adhesion and extravasation in postcapillary venules. We found that while neutrophil rolling velocity was decreased by 14% (± 4%), neutrophil adhesion increased significantly by 20% (± 6%) and extravasation by 20% (± 4.8%) in Csk^iECKO^ mice when compared to Csk^lox/lox^ mice (**Figures 3A–C**; illustratory videos: **Supplementary Videos 1**, **2**). Parameters such as the number of rolling neutrophils, rolling flux fraction, and hemodynamic parameters were not affected by Csk gene inactivation (**Supplementary Figures 1B–H**).

Next, we decided to verify these effects in *in vitro* transmigration assays. We isolated primary lung endothelial cells from Csk^iECKO^ and treated them in culture for 5 days with either tamoxifen or solvent alone. After stimulation with TNF-α for 4 h, we analyzed the adhesion of mouse neutrophils. We found that Csk gene inactivation caused an 18% (± 4.6%) increase in adherent neutrophils (**Figure 3D**). In separate assays, we cultured lung endothelial cells (ECs) from Csk^iECKO^ on Transwell filters, and we analyzed neutrophil transmigration toward a gradient of CXCL1. Again, we found that tamoxifen-induced Csk gene inactivation led to a 26% (± 2.6%) increase in neutrophil transmigration, reproducing our *in vivo* results (**Figure 3E**).

We have previously shown that Csk binds to the phosphorylated Y685 of VE-cadherin (35). Interestingly, knock-in mice expressing the point-mutated VE-cadherin-Y685F instead of WT VE-cadherin showed a similar slight but significant increase in neutrophil extravasation (26) as we show here for the Csk^iECKO^ mice. This raised the question of whether Y685 of VE-cadherin might be relevant for this effect. To answer this, we analyzed the transmigration of mouse neutrophils through primary isolated lung endothelial cells from VE-cadherin-Y685F/Csk^iECKO^ mice. We treated the endothelial cells prior to the assays with either solvent or tamoxifen. We found that even when Csk expression was unaffected, the Y685F mutation of VE-cadherin was sufficient to increase neutrophil transmigration efficiency by 21% (± 5.6%) (**Figure 3E**). Csk gene inactivation in the double-mutant endothelial cells had no significant additive, increasing effect on transmigration [28% (± 7.4%)] (**Figure 3E**). The efficiency of *in vitro* Csk gene inactivation was verified by immunoblotting endothelial cell lysates for Csk (**Figure 3F**). Collectively, these results suggest that Csk dampens neutrophil transmigration in a way that is linked to the presence of Y685 of VE-cadherin.

### Csk deficiency and the Y685F mutation of VE-cadherin increase the level of activated SFK at endothelial junctions

**Figure 4:**
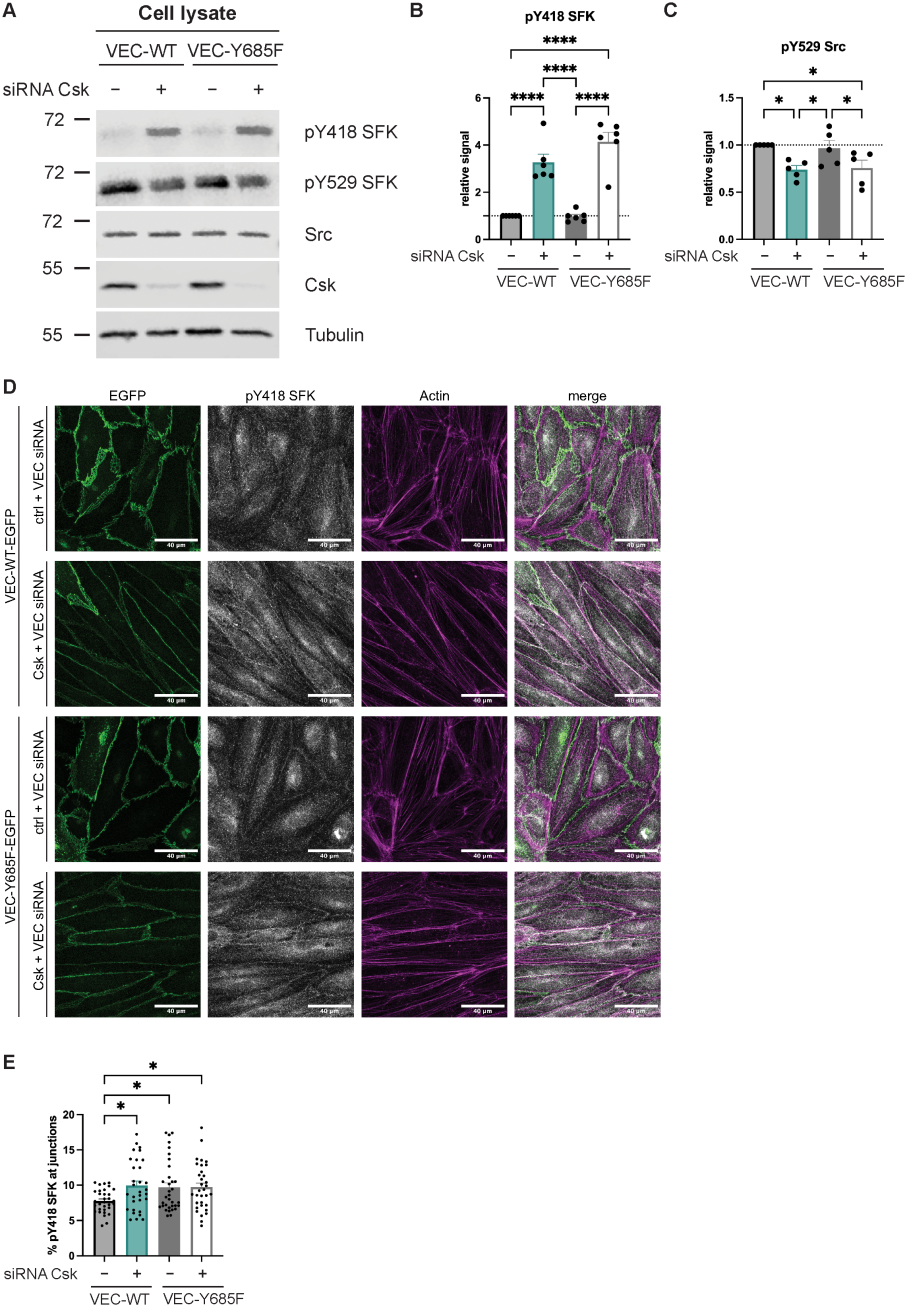
Csk deficiency and the Y685F VE-cadherin mutation increase local SFK activity at junctions. **(A–C)** Control or Csk siRNA-treated confluent human umbilical vein endothelial cells (HUVECs) were transfected with VE-cadherin-WT-EGFP or Y685F-EGFP adenovirus, lysed, and immunoblotted with antibodies against phosphorylated SFK-Y418, SFK-Y529, Src, Csk, and α-tubulin [**A,** representative of six (SFK-pY418) or five (SFK-pY529, Csk) independent experiments]. Molecular weight markers are indicated in kDa. The graphs depict the normalized relative Y418 SFK signal **(B)** or pY529 SFK signal **(C)** detected in the Western blots. The signal was normalized to total Src and α-tubulin and is presented relative to that of control cells, set as 1 [n = 6 **(B)**, n = 5 **(C)**, mean ± SEM, *p < 0.05, ****p < 0.0001, one-way ANOVA]. **(D, E)** Control or Csk siRNA-treated HUVECs were transfected with VE-cadherin WT-EGFP or VE-cadherin-Y685F-EGFP adenovirus. Subsequently, cells were fixed, permeabilized, and stained with antibodies against phosphorylated SFK-Y418 and actin (phalloidin) (**D,** representative of six independent experiments). Scale bars are 40 µm. The graph depicts the percentage of the SFK-Y418 signal detected at the junctions **(E)** as quantified using an ImageJ macro. One data point depicts the quantification from one image (n = 6, mean ± SEM, *p < 0.05, one-way ANOVA).

Next, we tested whether Y685 of VE-cadherin is relevant for the control of SFK activation by Csk. Since transduction efficiency was low in primary mouse endothelial cells, we switched to HUVECs for these experiments. We treated HUVECs with siRNA against VE-cadherin (targeting a site in the upstream coding region) and siRNA targeting Csk or ctrl siRNA. Subsequently (48 h later), we transduced cells with either VE-cadherin-WT-EGFP or VE-cadherin-Y685F-EGFP. As revealed by immunoblotting of cell lysates, silencing of Csk led to reduced phosphorylation of the inactivating Y529, whereas signals for the activating pY418 were increased. These effects were identical in HUVECs expressing WT or mutant VE-cadherin. Simply expressing VE-cadherin-Y685F in the presence of endogenous Csk had no significant effect on SFK activation as analyzed from cell lysates (**Figure 4A**). The quantification of the pY418 and pY529 signals are shown in **Figures 4B, C**. This argues for a general control function of Csk for the activation of total cellular SFKs, which goes beyond the relevance of VE-cadherin-pY685 as an anchor for Csk.

Then, the relevance of VE-cadherin-pY685 for the control of SFK activation by Csk at endothelial junctions was tested. HUVECs were silenced for endogenous VE-cadherin and treated with either Csk siRNA or ctrl siRNA, followed by transduction with VE-cadherin-WT-EGFP or VE-cadherin-Y685F-EGFP, as described above. Cells were analyzed by staining for SFK-pY418, EGFP, and actin. As shown in **Figure 4D**, silencing Csk increased the level of activated SFK at endothelial junctions. This was also achieved simply by expressing VE-cadherin-Y685F, even when endogenous Csk was still expressed (**Figure 4D**). The level of activated SFK was not further increased by Csk silencing in cells that expressed VE-cadherin-Y685F (**Figure 4D**). The quantification of junctional SFK-pY418 is shown in **Figure 4E**. These results suggest that VE-cadherin-Y685 is relevant for the regulation of SFK activity at junctions by Csk.

### Cortactin is required for the effect of Csk on leukocyte extravasation

**Figure 5:**
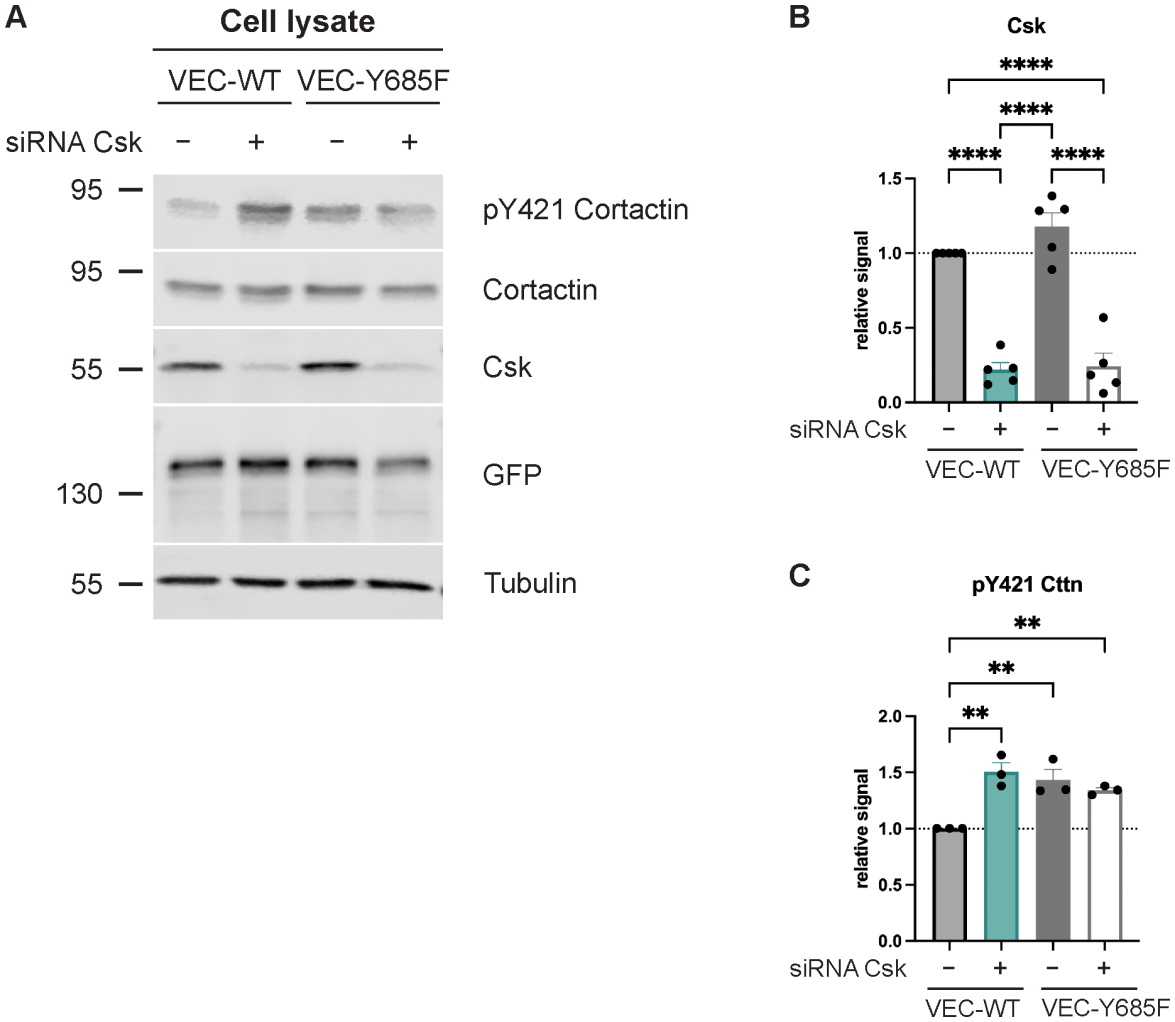
Csk deficiency as well as Y685F VE-cadherin mutation increase Cortactin phosphorylation levels. **(A)** Control or Csk siRNA-treated confluent human umbilical vein endothelial cells (HUVECs) were transfected with VE-cadherin-WT-EGFP or -Y685F-EGFP adenovirus, lysed, and immunoblotted with antibodies against phosphorylated Cortactin-Y421, Csk, GFP, and α-tubulin [representative of five (Csk) or three (pY421 Cttn) independent experiments]. Molecular weight markers are indicated in kDa. The graphs depict the normalized relative Csk **(B)** and Cttn phosphorylation **(C)** signals detected in the Western blots. The signal was normalized to total cortactin **(C)** and α-tubulin and is presented relative to that of control cells, set as 1 [n = 5 **(B)**, n = 3 **(C)**, mean ± SEM, **p < 0.01, ****p < 0.0001, one-way ANOVA].

Cortactin is one of the first identified Src substrates. We found previously that it is required in endothelial cells for neutrophil extravasation (17). Therefore, we tested whether cortactin is involved in the mechanism by which Csk controls neutrophil diapedesis. First, we analyzed whether silencing of Csk in HUVECs would increase the phosphorylation of an activating tyrosine residue of cortactin and whether the Csk binding site of VE-cadherin would be relevant for this effect. HUVECs were silenced for endogenous VE-cadherin and treated with either Csk siRNA or ctrl siRNA, followed by transduction with WT VE-cadherin-EGFP or VE-cadherin-Y685F-EGFP, as described above. By immunoblotting cell lysates, we analyzed the pY421 levels of cortactin as well as the expression levels of cortactin, Csk, tubulin, and EGFP-fusion protein (**Figure 5A**). The quantification of the Csk blot signals is shown in **Figure 5B** and that of the pY421 activation epitope of cortactin in **Figure 5C**. We found that silencing of Csk increased pY421 levels of cortactin. Similarly, the expression of VE-cadherin-Y685F was sufficient to increase cortactin-pY421 levels even when Csk was expressed at endogenous levels. Silencing of Csk in cells expressing the VE-cadherin mutant had no additional stimulating effect on cortactin phosphorylation. These results suggest that Csk limits the activation level of cortactin, and the need for Y685 of VE-cadherin for this effect suggests that Csk recruitment to pY685 of VE-cadherin is involved in the regulation of cortactin activity.

**Figure 6:**
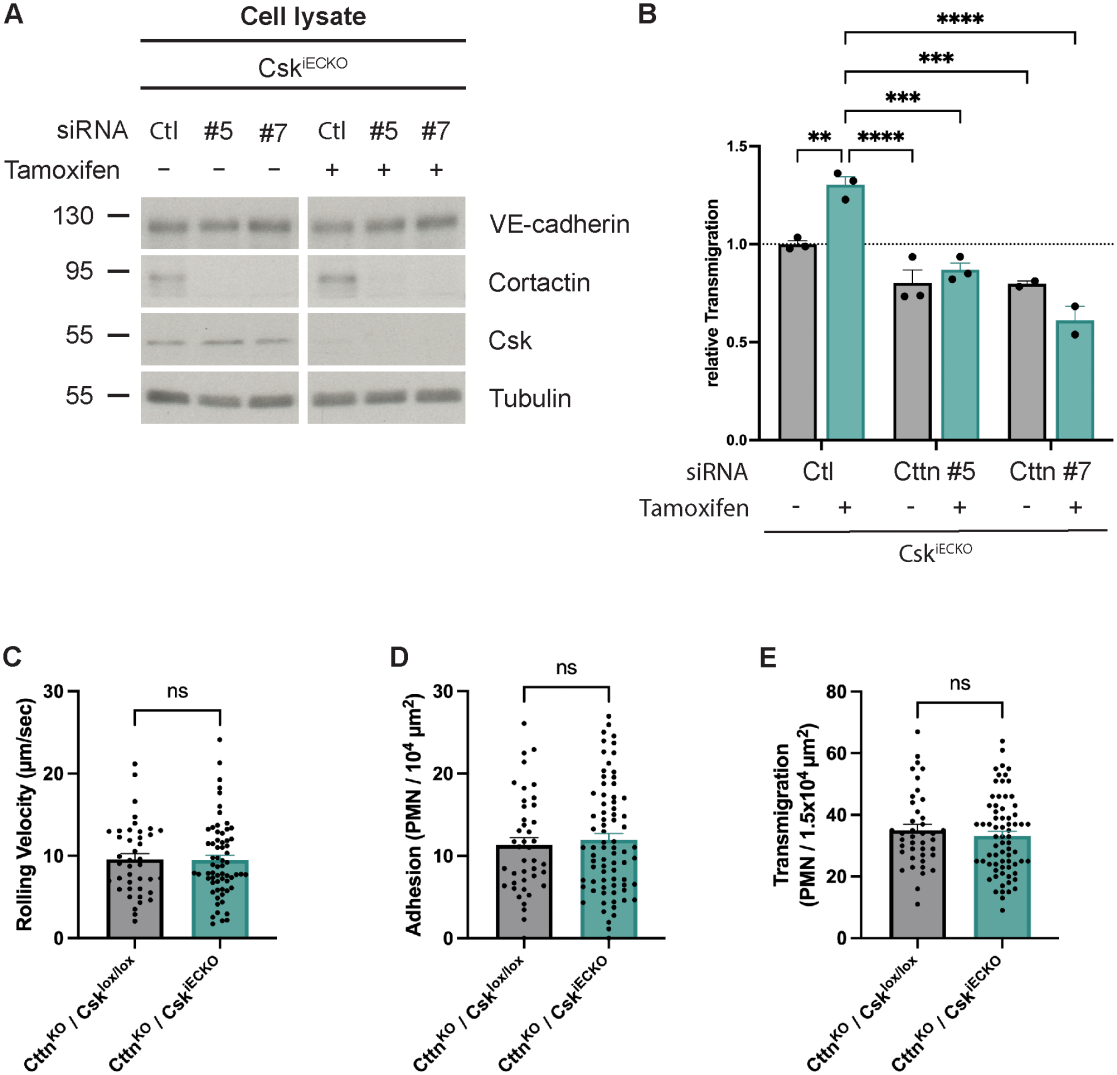
Csk-dependent increase in leukocyte transmigration requires the presence of cortactin *in vitro* and *in vivo*. **(A)** Lung endothelial cells from Csk^iECKO^ mice treated with solvent (−) or tamoxifen (+) (as indicated) were electroporated with scrambled siRNA or cortactin (Cttn) siRNA (two different sequences). Total cell lysates were immunoblotted for VE-cadherin, cortactin, Csk, and α-tubulin (representative of three independent experiments). Molecular weight markers are indicated in kDa. **(B)** Lung endothelial cells from Csk^iECKO^ mice treated with either solvent or tamoxifen were electroporated with scrambled siRNA or cortactin (Cttn) siRNA (two different sequences). Cells were subsequently seeded on Transwell filters, and after 48 h, the monolayer was stimulated with TNF-α, and murine neutrophils were allowed to transmigrate toward the chemokine CXCL-1. The transmigration rate is presented relative to that of control cells, which was set as 1. One data point represents the mean transmigration determined in one experiment [n = 3 (siRNA Ctrl and siRNA Cttn #5) or n = 2 (siRNA Cttn #7), mean ± SEM, **p < 0.01, ***p < 0.001, ****p < 0.0001, two-way ANOVA]. **(C–E)** Cttn^KO^/Csk^lox/lox^ and Cttn^KO^/Csk^iECKO^ mice were i.p. injected with tamoxifen daily for 5 days. The graphs depict rolling velocity **(C)**, adherent **(D)**, and extravasated **(E)** leukocytes in cremaster tissue of mice stimulated intrascrotally with IL-1β for 4 h before intravital microscopy (Cttn^KO^/Csk^lox/lox^: n = 7 mice, n = 42 vessels; Cttn^KO^/Csk^iECKO^: n = 11 mice, n = 77 vessels; mean ± SEM, t-test).

Next, we tested whether the effect of Csk on neutrophil diapedesis depends on the regulation of cortactin activity. To this end, we treated primary lung endothelial cells from Csk^iECKO^ mice with tamoxifen or solvent for 5 days, followed by siRNA treatment with either two different siRNAs for cortactin or with control siRNA. We verified the efficiency of *in vitro* Csk gene inactivation and cortactin siRNA treatment by immunoblotting the endothelial cells for Csk (**Figure 6A**). We then analyzed the CXCL1-stimulated migration of mouse neutrophils through TNF-α-activated monolayers of these cells. In this way, we found that Csk gene inactivation increased neutrophil transmigration when the endothelial cells expressed normal cortactin levels; however, this increase was blocked when cortactin expression was silenced (**Figure 6B**). Thus, Csk modulates neutrophil transmigration through a mechanism that requires cortactin.

To verify this *in vitro* finding *in vivo*, we analyzed neutrophil extravasation by intravital microscopy of the cremaster muscle of tamoxifen-treated double-mutant cortactin^−/−^/Csk^lox/lox^ and cortactin^−/−^/Csk^iECKO^ mice after i.s. stimulation with IL-1β 4 h before analysis (illustratory videos: **Supplementary Videos 3**, **4**). Hemodynamic and leukocyte rolling parameters are shown in **Supplementary Figure 2** and were not affected by gene inactivation of Csk. We found that neutrophil rolling velocity, adhesion, and neutrophil extravasation were not significantly increased by gene inactivation of Csk in cortactin^−/−^ mice (**Figures 6C–E**). This was in contrast to our results with mice that expressed cortactin (**Figures 3A–C**). Thus, cortactin is required for the effect of Csk on neutrophil extravasation *in vivo*.

### The increase in neutrophil transmigration triggered by Csk gene inactivation requires functional ICAM-1

**Figure 7:**
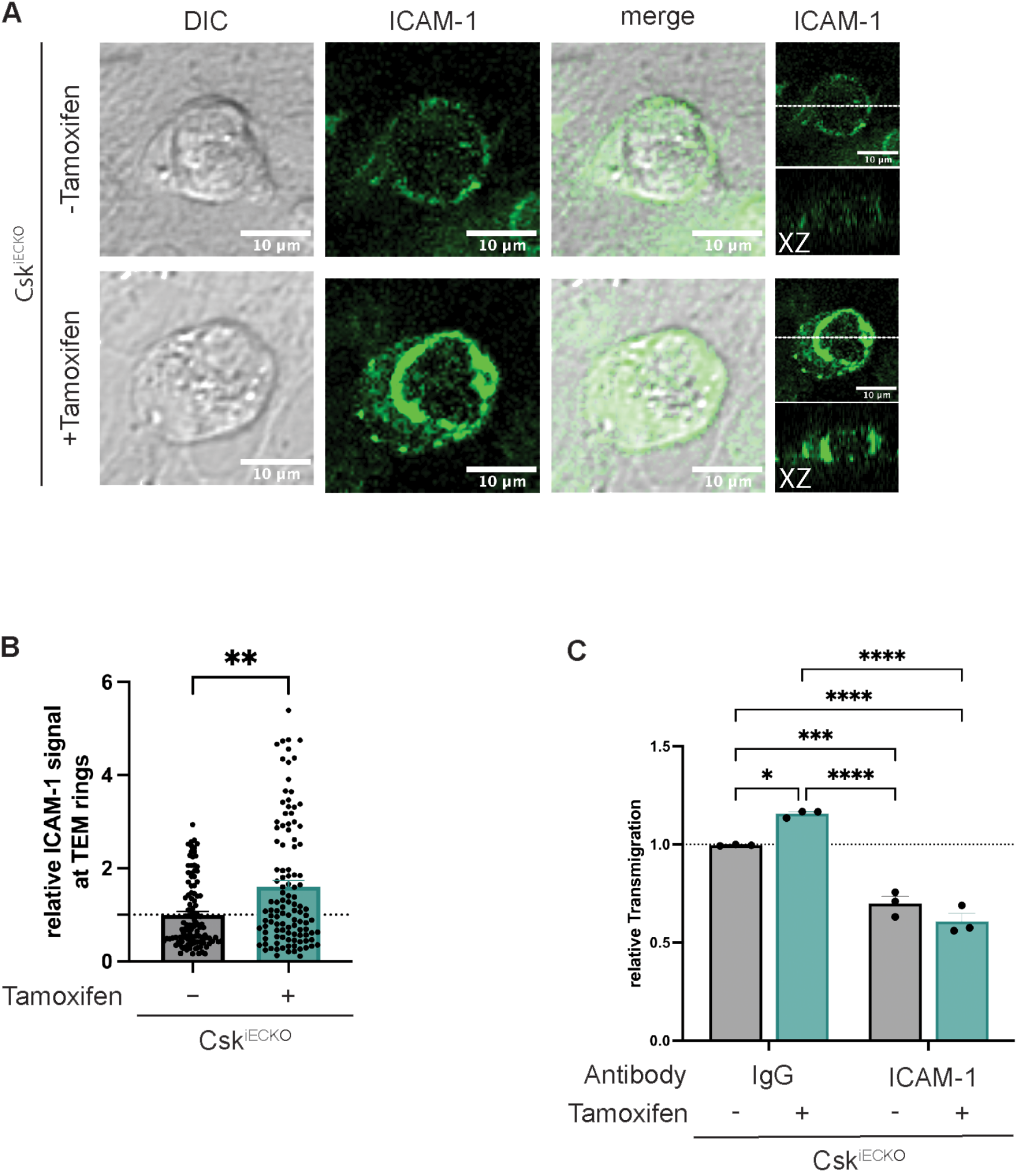
Increased leukocyte transmigration after Csk knock-out is mediated by higher ICAM-1 recruitment to adherent leukocytes. **(A, B)** Endothelial cells were isolated from the lungs of Csk^iECKO^ mice, treated with solvent (−) or tamoxifen (+) (as indicated), and grown to confluence on fibronectin-coated slides. After overnight stimulation with TNF-α, the cells were incubated with 1 × 10^6^ polymorphonuclear neutrophils (PMNs) from wild-type (WT) mice for 15 min. Non-adherent leukocytes were washed away; cells were fixed, permeabilized, and stained for ICAM-1. **(A)** Confocal XY images of endothelial cell ICAM-1 and DIC show clustering of ICAM-1 around a transmigrating neutrophil. Side view projections (X–Z) are depicted on the right. The horizontal white line in the last panel indicates the position of the X–Z slice. **(B)** The graphs depict the relative mean fluorescence intensity quantified in the z-slice depicting the ring-like ICAM-1 structure around transmigrating neutrophils (n = 4 experiments, 114 ICAM-1 ring-like structures per condition, mean ± SEM, **p < 0.01, t-test). **(C)** Transmigration of murine neutrophils toward the chemokine CXCL-1 through a TNF-α-stimulated monolayer of either solvent- (−) or tamoxifen-treated (+) endothelial cells isolated from the lungs of Csk^iECKO^ mice. Cells were pretreated with isotype control or anti-ICAM-1 (YNI.1) antibodies for 30 min at 37°C prior to the addition of neutrophils. The transmigration rate is presented relative to that of control cells, which was set as 1. One data point represents the mean transmigration determined in one experiment (n = 3, mean ± SEM, *p < 0.05, ***p < 0.001, ****p < 0.0001, two-way ANOVA).

Since we and others have shown before that cortactin supports the recruitment and clustering of ICAM-1 around transmigrating neutrophils (14, 17) and that this requires tyrosine phosphorylation of cortactin (15), we tested whether gene inactivation of Csk would affect ICAM-1 interaction with neutrophils. To this end, we treated primary mouse lung endothelial cells from Csk^iECKO^ mice with tamoxifen or solvent, exposed them overnight to TNF-α, and allowed mouse PMNs to adhere and transmigrate. We stained fixed cells for ICAM-1, and we evaluated the intensity of ICAM-1 signals surrounding neutrophils. A representative picture of ICAM-1 clusters surrounding neutrophils is shown in **Figure 7A**. Note that the ICAM-1 structures supported by Csk gene inactivation resembled “docking structures” or “transmigratory cups” as they were described for transmigrating leukocytes before (8, 9). The quantification of 114 ICAM-1 positive ring-like structures for each condition revealed a more than 50% increase in ICAM-1 signal intensity around neutrophils that interacted with endothelial cells gene-inactivated for Csk (**Figure 7B**). In agreement with this, we found that mouse neutrophil transmigration through monolayers of mouse primary lung endothelial cells grown on Transwell filters increased upon Csk gene inactivation. This increase was not detected anymore when ICAM-1 was blocked with an anti-ICAM-1 antibody (**Figure 7C**). Thus, Csk gene inactivation augments ICAM-1 clustering around neutrophils and supports increased neutrophil diapedesis in an ICAM-1-dependent way.

## Discussion

**Figure 8:**
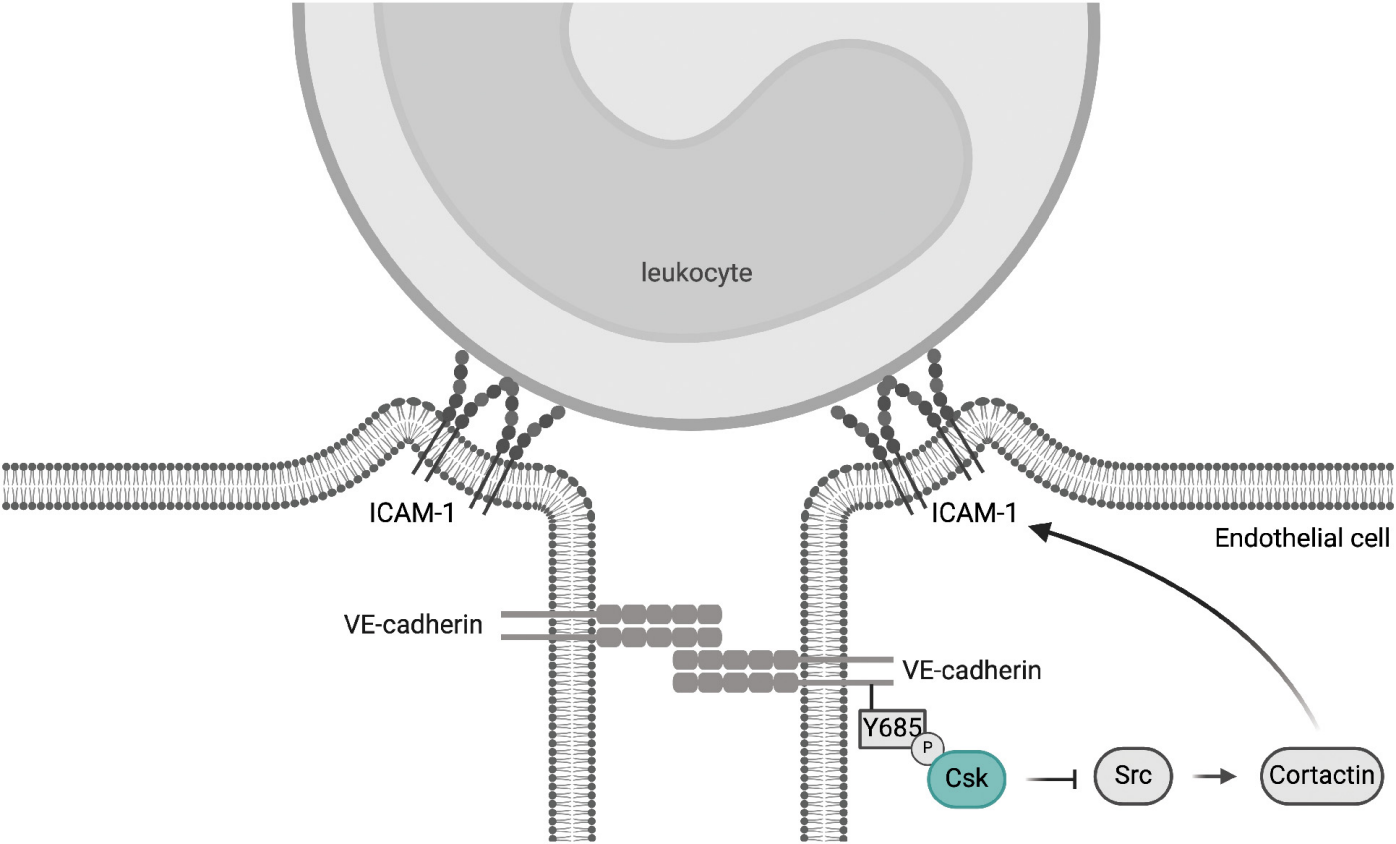
Proposed mechanism of Csk-mediated attenuation of the leukocyte extravasation process. Inflammation-related phosphorylation of VE-cadherin Y685 initiates a signaling mechanism that inhibits excessive leukocyte diapedesis by recruiting Csk to cell–cell junctions, thereby controlling junctional SFK activity. This in turn restricts cortactin phosphorylation and thereby ICAM-1 clustering and neutrophil extravasation. This mechanism may explain how the phosphorylation of VE-cadherin-Y685 can dampen the extravasation of leukocytes, although at the same time phosphorylation of this site destabilizes junctions and induces vascular leaks. Graphical abstract created with BioRender.com [Holstein, K., Stegmeyer, R. (2024) BioRender.com/s88p818].

In this study, we investigated whether Csk is relevant for the control of endothelial barrier integrity. We found that the deficiency of Csk in endothelial cells leads to the activation of SFKs and phosphorylation of Y685 of VE-cadherin, but it does not affect baseline vascular permeability or inflammation-induced vascular leaks. In contrast to this lack of a net effect on vascular permeability, we detected a significant increase in neutrophil extravasation when Csk was depleted. Mechanistically, this effect required *in vitro* and *in vivo* the presence of cortactin and was abolished by blocking ICAM-1. In line with this, Csk depletion increased tyrosine phosphorylation of the SFK substrate cortactin and augmented the clustering of endothelial ICAM-1 around interacting neutrophils, which are phenomena known to support the diapedesis process. Interestingly, the Csk binding site Y685 of VE-cadherin was needed for the regulatory effect of Csk on SFK activation at junctions, phosphorylation of cortactin, and neutrophil diapedesis. This provides an explanation for why the VE-cadherin-Y685F mutation augments neutrophil adhesion to and diapedesis through the endothelial cell barrier. Collectively, our findings suggest that VE-cadherin-Y685 phosphorylation triggers a negative feedback loop that curbs leukocyte extravasation by recruiting Csk, which attenuates SFK activity and cortactin phosphorylation, which in turn restricts endothelial ICAM-1 clustering around neutrophils and neutrophil diapedesis (**Figure 8**).

Tyrosine 685 of VE-cadherin has been reported to be a major and direct substrate for Src within the cytoplasmic domain of VE-cadherin (22). In addition, it is well established that point-mutated VE-cadherin-Y685F knock-in mice show reduced induction of vascular permeability in various inflammation models in the skin (26), cremaster muscle (27), and retina (28). Therefore, at first glance, it was surprising that gene inactivation of Csk, which leads to SFK activation and upregulation of VE-cadherin-Y685 phosphorylation, had no effect on baseline vascular permeability or the induction of vascular leaks in inflammation. However, this apparent discrepancy is less surprising when one considers that Csk counteracts the activation of several SFKs and that each of these SFKs has numerous substrates. Endothelial cells mainly express the three SFKs, namely, Src, Yes, and Fyn (44). Although it was previously reported that gene inactivation of Src and Yes each reduced VEGF-stimulated vascular leaks in mouse skin (21), a recent thorough analysis of mice with endothelial-specific gene deletions for Yes or Src revealed that Src and Yes have opposite effects on endothelial junction integrity (29). Src destabilized junctions, whereas Yes supported junction integrity. Each kinase supported the phosphorylation of VE-cadherin-Y685. Thus, in contrast to Src, the effect of Yes on VE-cadherin-Y685 phosphorylation did not correlate with its effect on junction integrity.

This is not in conflict with the role of VE-cadherin-Y685 phosphorylation as a supporter of vascular permeability since the Y685F mutation indeed clearly inhibits permeability induction, but this inhibition is not complete. In fact, permeability induction in the skin by VEGF and histamine is significantly but only partially inhibited by 35% in VE-cadherin-Y685F mice (26). Thus, there is still significant leak formation in mice in the absence of any Y685 phosphorylation of VE-cadherin. Similarly, overexpression of Yes stimulated VE-cadherin-Y685 phosphorylation but did not destabilize junctions (29). Again, Yes appears to drive mechanisms that override the destabilizing effect of Y685 phosphorylation. While it is not known what these mechanisms are, we have shown previously that Tie-2-driven mechanisms exist that stabilize endothelial junction integrity *in vivo* even when VE-cadherin is phosphorylated (45). Thus, VE-cadherin-Y685 phosphorylation is an important but, of course, not the only regulatory mechanism for endothelial junction integrity.

Our finding that endothelial-specific Csk gene inactivation has no net influence on vascular permeability in the skin of mice is in agreement with an *in vitro* study that showed that overexpressing dominant negative Csk in HUVECs did not interfere with junction integrity, although it caused an upregulation of Y685 phosphorylation levels of VE-cadherin (46). Again, these results may be related to the fact that dampening of Csk activity would be expected to stimulate opposite effects on junction integrity due to the simultaneous activation of Src and Yes.

As we have shown previously, VE-cadherin function and endothelial junction integrity are differently regulated during leukocyte diapedesis and plasma leak induction, with Y731 being selectively required for the regulation of leukocyte extravasation and Y685 exclusively for the regulation of plasma leak (26). While the signaling mechanisms downstream of each tyrosine residue are different, they lead in each case to a reduction in endothelial junction integrity (7, 40). In the context of these selective mechanisms, it was surprising and mechanistically unexplained that the Y685F mutation of VE-cadherin augmented neutrophil extravasation (26). Here, we have found the underlying mechanism. In contrast to the role of Y685 phosphorylation as an inducer of vascular plasma leaks, its role in leukocyte extravasation is independent of junction integrity. Instead, phosphorylation of VE-cadherin-Y685 counteracts leukocyte extravasation by inhibiting cortactin and ICAM-1 function and thereby leukocyte docking and diapedesis, which is why the Y685F mutation augments the process.

The need for two different mechanisms by which Y731 and Y685 phosphorylation of VE-cadherin influence endothelial junction destabilization may be related to the fact that the passage of leukocytes requires larger gaps at endothelial junctions than does the passage of plasma proteins. Indeed, it has been shown in an allergy-induced inflammation model that leukocyte extravasation and vascular leak formation occur at different sites in the vasculature of the trachea (47). Our novel findings presented here establish an additional mechanism that ensures that Y685 phosphorylation not only exclusively induces plasma leak formation but also actively prevents or at least reduces leukocyte extravasation. Thus, it appears that endothelial mechanisms exist that ensure selectivity at certain vascular sites for plasma leak formation in the absence of leukocyte extravasation.

SFKs are anchored to the membrane by their N-terminal myristate and palmitate moieties. In contrast, Csk lacks fatty acyl modifications that could link it to the plasma membrane. Therefore, cytosolic Csk needs to be recruited to the membrane by scaffolding membrane proteins. Generally, the Csk binding protein (Cbp/PAG1) is a ubiquitously expressed major regulator of Csk that provides a phosphorylated tyrosine residue for binding of the SH2 domain of Csk (48, 49). This activates Csk and recruits it to sites where it is needed. In addition to this important Csk scaffolding protein, other membrane proteins have been described that can also serve as recruiting devices for Csk (35, 50–56). One of them is VE-cadherin, which we have shown previously to recruit Csk to endothelial cell junctions by binding of its phosphorylated Y685 to the Csk-SH2 domain (35). In light of the various known alternative scaffolding proteins of Csk, it is remarkable that it is VE-cadherin-pY685 that serves as the essential scaffold, which is responsible for Csk-mediated regulation of junctional SFK activation, cortactin phosphorylation, and cortactin-supported neutrophil diapedesis. This explains why neutrophil extravasation is augmented in VE-cadherin-Y685F mutant mice.

Cortactin is one of the first identified Src substrates, and its tyrosine phosphorylation is needed for the support of neutrophil diapedesis and ICAM-1 clustering around transmigrating neutrophils (14, 15). The contribution of endothelial cortactin to leukocyte transmigration was also related to F-actin dynamics, which affected the mobility of cortactin in its interactions with ICAM-1 (16). We found later that cortactin is also relevant for neutrophil extravasation *in vivo* and showed mechanistically that cortactin supports ICAM-1 clustering by contributing to the temporal and spatial regulation of RhoG (17). While these studies demonstrated how cortactin supports neutrophil extravasation, the present study reports a novel mechanism that negatively influences this contribution of cortactin and links it to the phosphorylation of VE-cadherin Y685.

The endothelium at different sites of the vascular tree and in different organs is heterogeneous. Our study focused on primary endothelial cells from mouse lungs and the human umbilical vein and investigated *in vivo* leukocyte extravasation in the cremaster muscle, one of the best studied organs for this process. Although mechanistic differences between the endothelia of these different origins are likely, it is remarkable that the contribution of Csk and cortactin to leukocyte transmigration appears to be conserved.

In summary, we reported here a signaling mechanism that is based on the recruitment of Csk to VE-cadherin-Y685, which controls junctional SFK activity and thereby cortactin-mediated support of ICAM-1 function and leukocyte diapedesis. This mechanism explains how the phosphorylation of VE-cadherin-Y685 can dampen the extravasation of leukocytes, although at the same time phosphorylation of this site destabilizes junctions and induces vascular leaks. We concluded that cellular mechanisms exist that trigger inflammation-induced plasma leaks and, at the same time and location, inhibit the infiltration of neutrophils.

## Data Availability

The raw data supporting the conclusions of this article will be made available by the authors, without undue reservation.

## Supplementary material

Supplementary Figure 1
**(A)** Generation of conditional Csk knock-out mice. The mice were generated by breeding Csk^lox/lox^ mice (36) with mice expressing a tamoxifen-inducible form of the Cre recombinase under the transcriptional control of the pdgfb gene (37). Numbered rectangles represent exons. **(B)** Csk^lox/lox^ and Csk^iECKO^ mice were i.p. injected with tamoxifen daily for 5 days. The graphs depict vessel diameter **(A)**, total leukocyte number **(B)**, leukocyte velocity **(C)**, rolling number of PMNs **(D)**, rolling flux fraction **(E)**, wall shear rate **(F)**, and Newtonian wall shear rate **(G)** in cremaster tissue from mice stimulated intrascrotally with IL-1β for 4 h before intravital microscopy (Csk^lox/lox^: n=6 mice, n=57 vessels; Csk^iECKO^: n=5 mice, n=43 vessels; mean ± SEM, *p<0,05, **p <0,01, t-test).

Supplementary Figure 2
**(A–G)** Cttn^KO^/Csk^lox/lox^ and Cttn^KO^/Csk^iECKO^ mice were i.p. injected with tamoxifen daily for 5 days. The graphs depict vessel diameter **(A)**, total leukocyte number **(B)**, blood flow velocity **(C)**, rolling number of PMNs **(D)**, rolling flux fraction **(E)**, wall shear rate **(F)**, and Newtonian wall shear rate **(G)** in cremaster tissue from mice stimulated intrascrotally with IL-1β for 4 h before intravital microscopy (For vessel diameter and total leukocyte number: Cttn^KO^/Csk^lox/lox^: n=7 mice, n=42 vessels; Cttn^KO^/Csk^iECKO^: n=11 mice, n=77 vessels; mean ± SEM, t-test. For all other parameters: Cttn^KO/^Csk^lox/lox^: n=3 mice, n=23 vessels; Cttn^KO/^Csk^iECKO^: n=4 mice, n=23 vessels; mean ± SEM, t-test).

Supplementary Video 1 Intravital microscopy of an inflamed cremaster muscle venule of a Csk^lox/lox^ animal. Csk^lox/lox^ mice were i.p. injected with tamoxifen daily for 5 days. The illustrative time-lapse video of a postcapillary venule was recorded in cremaster tissue using an upright Axio Examiner microscope at a rate of 10 frames/s.

Supplementary Video 2 Intravital microscopy of an inflamed cremaster muscle venule of a Csk^iECKO^ animal. Csk^iECKO^ mice were i.p. injected with tamoxifen daily for 5 days. The illustrative time-lapse video of a postcapillary venule was recorded in cremaster tissue using an upright Axio Examiner microscope at a rate of 10 frames/s.

Supplementary Video 3 Intravital microscopy of an inflamed cremaster muscle venule of a Cttn^KO^/Csk^lox/lox^ animal. Cttn^KO^/Csk^lox/lox^ mice were i.p. injected with tamoxifen daily for 5 days. The illustrative time-lapse video of a postcapillary venule was recorded in cremaster tissue using an upright Axio Examiner microscope at a rate of 10 frames/s.

Supplementary Video 4 Intravital microscopy of an inflamed cremaster muscle venule of a Cttn^KO^/Csk^iECKO^ animal. Cttn^KO^/Csk^iECKO^ mice were i.p. injected with tamoxifen daily for 5 days. The illustrative time-lapse video of a postcapillary venule was recorded in cremaster tissue using an upright Axio Examiner microscope at a rate of 10 frames/s.
