# Supplementary figures and images for: Csk controls leukocyte extravasation via local regulation of Src family kinases and cortactin signaling
Source: Front Immunol. 2024 Oct 28;15:1480152. doi: 10.3389/fimmu.2024.1480152 (PMC11550946; doi:10.3389/fimmu.2024.1480152)

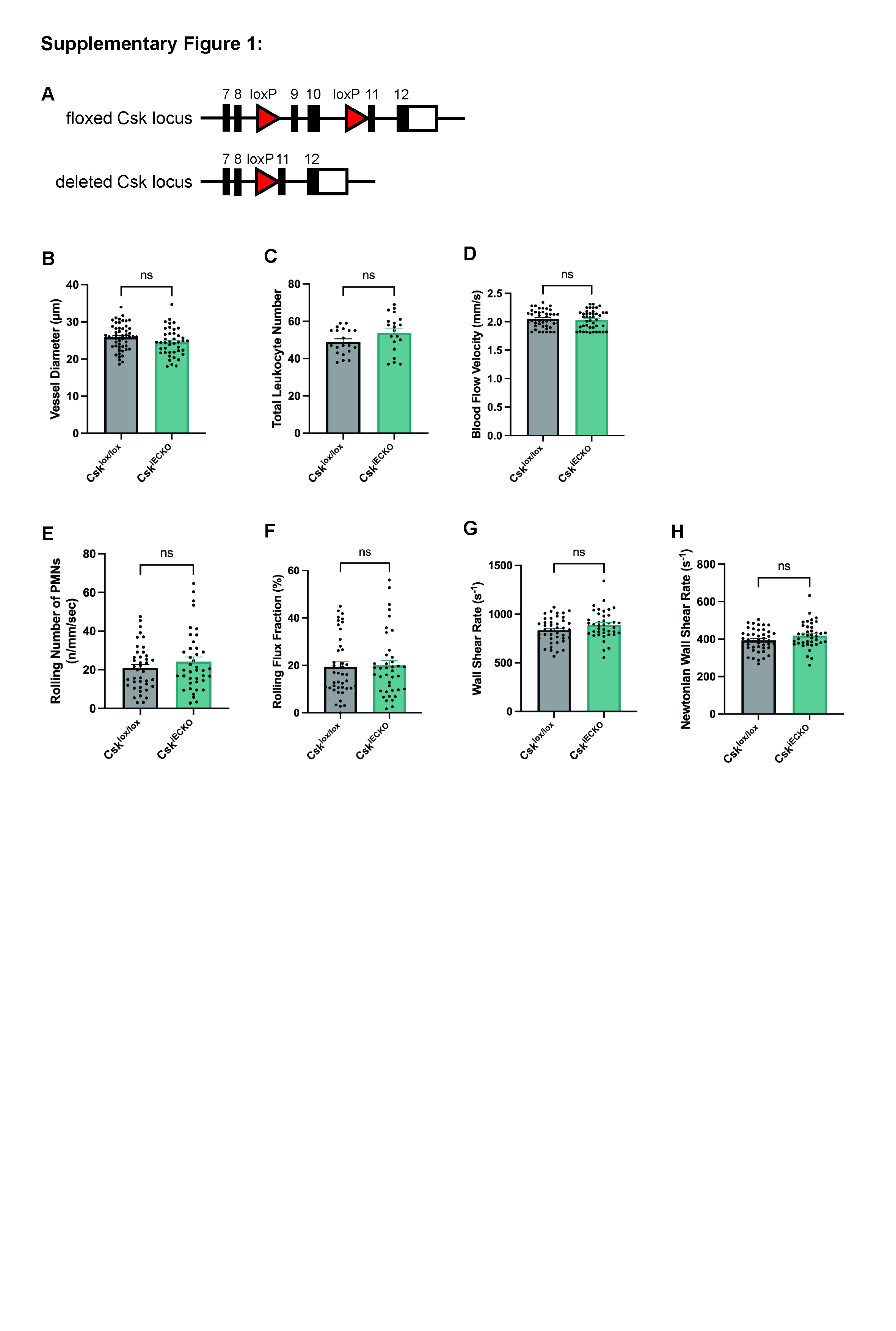

Supplement: Supplementary Figure 1 — (A) Generation of conditional Csk knock-out mice. The mice were generated by breeding Csklox/lox mice (36) with mice expressing a tamoxifen-inducible form of the Cre recombinase under the transcriptional control of the pdgfb gene (37). Numbered rectangles represent exons. (B) Csklox/lox and CskiECKO mice were i.p. injected with tamoxifen daily for 5 days. The graphs depict vessel diameter (A), total leukocyte number (B), leukocyte velocity (C), rolling number of PMNs (D), rolling flux fraction (E), wall shear rate (F), and Newtonian wall shear rate (G) in cremaster tissue from mice stimulated intrascrotally with IL-1β for 4 h before intravital microscopy (Csklox/lox: n=6 mice, n=57 vessels; CskiECKO: n=5 mice, n=43 vessels; mean ± SEM, *p<0,05, **p <0,01, t-test). [file Image1.tif]

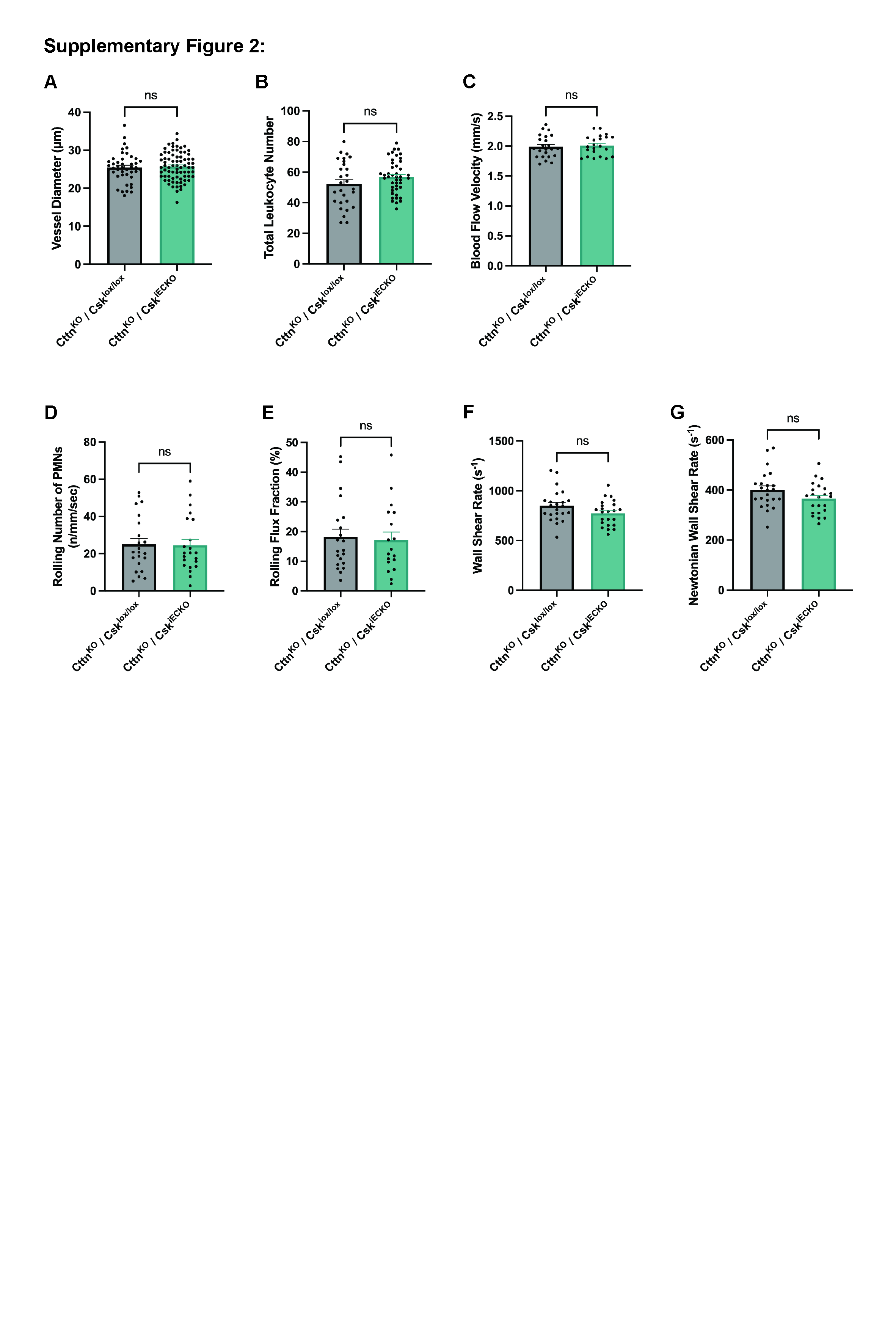

Supplement: Supplementary Figure 2 — (A–G) CttnKO/Csklox/lox and CttnKO/CskiECKO mice were i.p. injected with tamoxifen daily for 5 days. The graphs depict vessel diameter (A), total leukocyte number (B), blood flow velocity (C), rolling number of PMNs (D), rolling flux fraction (E), wall shear rate (F), and Newtonian wall shear rate (G) in cremaster tissue from mice stimulated intrascrotally with IL-1β for 4 h before intravital microscopy (For vessel diameter and total leukocyte number: CttnKO/Csklox/lox: n=7 mice, n=42 vessels; CttnKO/CskiECKO: n=11 mice, n=77 vessels; mean ± SEM, t-test. For all other parameters: CttnKO/Csklox/lox: n=3 mice, n=23 vessels; CttnKO/CskiECKO: n=4 mice, n=23 vessels; mean ± SEM, t-test). [file Image2.tif]
